# 重楼皂苷Ⅶ对弥漫大B细胞淋巴瘤细胞增殖、凋亡及细胞周期的影响

**DOI:** 10.3760/cma.j.cn121090-20230831-00099

**Published:** 2024-04

**Authors:** 雨晴 孙, 瑶 伏, 藕霄 纪, 丽娟 王

**Affiliations:** 1 锦州医科大学研究生培养基地（临沂市人民医院），临沂 276000 Graduate Training Base of Jinzhou Medical University（Linyi People's Hospital）, Linyi 276000, China; 2 临沂市人民医院中心实验室，临沂市肿瘤生物学重点实验室，临沂 276000 Linyi Key Laboratory of Tumor Biology, Linyi People's Hospital, Linyi 276000, China; 3 临沂市人民医院血液内科，徐州医科大学肿瘤转化医学重点实验室培育建设点，临沂 276000 Department of Hematology, Linyi People's Hospital, Key Laboratory of Tumor Translational Medicine, Xuzhou Medical University, Linyi 276000, China

## Abstract

探究重楼皂苷Ⅶ（PPⅦ）对弥漫大B细胞淋巴瘤（DLBCL）细胞株U2932和SUDHL-4的增殖、凋亡和细胞周期的影响。实验将DLBCL细胞株分为对照组和PPⅦ组，并使用MTT法、流式细胞术和Western blot法进行实验。结果显示，与对照组相比，PPⅦ显著抑制了U2932和SUDHL-4细胞的增殖（*P*<0.05）。细胞凋亡实验表明，0.5和1 µmol/L的PPⅦ处理使得两种细胞的凋亡率显著增加（*P*<0.05），并且凋亡相关蛋白的表达上调，而Bcl-2蛋白水平显著降低（*P*<0.05）。细胞周期实验显示，PPⅦ处理使得G0/G1期细胞增加（*P*<0.05），G2/M期细胞减少（*P*<0.05），且Cyclin D1、CDK4、CDK6和Survivin蛋白的表达量明显下调（*P*<0.05）。综上所述，PPⅦ通过抑制DLBCL细胞的增殖、促进细胞凋亡以及阻滞细胞于G0/G1期，发挥了抗淋巴瘤的作用。

## Abstract (en)

The aim of this study was to investigate the effects of polyphyllin Ⅶ（PPⅦ）on proliferation, apoptosis, and cell cycle of diffuse large B-cell lymphoma（PLBCL）cell lines U2932 and SUDHL-4. The DLBCL cell lines were divided into a control group and a PPⅦ group, and experiments were conducted using MTT assay, flow cytometry, and Western blotting. Results showed that compared with the control group, PPⅦ significantly inhibited the proliferation of U2932 and SUDHL-4 cells（*P*<0.05）. Apoptosis assays demonstrated that treatment with 0.50 and 1.00 µmol/L PPⅦ significantly increased the apoptosis rates of both cell lines（*P*<0.05）, upregulated apoptosis-related proteins, and downregulated Bcl-2 protein level（*P*<0.05）. Cell cycle analysis revealed that PPⅦ treatment led to an increase in G0/G1-phase cells（*P*<0.05）and a decrease in G2/M-phase cells（*P*<0.05）, significantly downregulated cyclin D1, CDK4, CDK6, and survivin protein expression（*P*<0.05）. In conclusion, PPⅦ exerted anti-lymphoma effects by inhibiting proliferation, promoting apoptosis, and inducing G0/G1 phase arrest in DLBCL cells.

弥漫大B细胞淋巴瘤（Diffuse large B-cell lymphoma，DLBCL）是非霍奇金淋巴瘤中最常见的一种类型，约占非霍奇金淋巴瘤的30％，患者通常表现为单个或多个淋巴结进行性肿大、结外疾病或两者兼有[1]–[2]。一线治疗方案R-CHOP（利妥昔单抗、环磷酰胺、多柔比星、长春新碱、泼尼松）方案能够治愈超过60％的DLBCL患者，但仍有部分复发或难治性患者，治疗效果差强人意，因此，迫切需要开发新的有效且不良反应少的治疗方案[3]–[4]。

重楼为云南重楼或七叶一枝花的干燥根茎，其功效包括清热解毒、镇静止痛、止血、消炎抑菌等。在现代研究中，已经探索了其各种新的药理活性，包括免疫调节、抗肿瘤、抗寄生虫等[5]，重楼皂苷Ⅶ（Polyphyllin Ⅶ，PPⅦ）是一种提取自重楼的甾体皂苷类活性成分，已有研究表明PPⅦ能够抑制胰腺癌PANC-1细胞增殖、迁移和侵袭，并可能通过下调PD-L1表达诱导PANC-1细胞凋亡，PPⅦ可抑制结肠癌SW-480细胞增长，诱导其凋亡并阻滞细胞周期于G0/G1期，进而发挥抗肿瘤作用[6]–[7]。但其对DLBCL的作用及机制研究较少，因此，本研究拟通过体外实验探讨PPⅦ对DLBCL细胞株SUDHL-4、U2932增殖、凋亡及细胞周期的影响，为DLBCL的治疗提供新的方向和选择。

## 材料与方法

1. 细胞来源及细胞培养：人DLBCL细胞株U2932和SUDHL-4均购自中国科学院上海细胞库，细胞培养条件为完全培养基，即含有1％双抗（青霉素-链霉素）、10％胎牛血清（FBS）的1640培养基，孵育于饱和湿度、37 °C和体积分数5％ CO_2_的细胞培养箱中，显微镜下观察细胞情况，待细胞密度为80％左右进行传代培养，取对数生长期且生长状态良好的细胞进行实验。

2. 药品与试剂：PPⅦ，购自上海源叶生物有限公司，溶解于二甲基亚砜（DMSO）中制成浓度为50 mmol/L原液于−80 °C冰箱储存，根据实验需要用完全培养基稀释至目标浓度，并保证DMSO的终浓度低于0.05％，使其不会对研究产生显著影响；FBS购自美国Gibco公司，MTT粉末、青霉素-链霉素和磷酸缓冲盐溶液（PBS）均购自北京索莱宝科技有限公司，Annexin Ⅴ-FITC细胞凋亡检测试剂盒、细胞裂解液及细胞周期检测试剂盒均购自中国贝博公司，PVDF膜购自美国Millipore公司，抗体GAPDH（60004-1-Ig）购自美国Proteintech公司，抗体Bax、Bcl-2、Smac、Cleaved Caspase 3、Cleaved Caspase 7、Cleaved Caspase 9、P53、Cyclin D1、CDK4、CDK6、Survivin（2772S、15071S、15108S、9664S、9491S、9505S、2527S、55506S、12790S、13331S、2808S）均购自美国Cell Signaling Technology公司，HRP-anti-mouse-IgG（HA1006）抗体、HRP-anti-rabbit-IgG（HA1001）抗体购自华安生物科技有限公司。

3. MTT法检测细胞增殖：取U2932、SUDHL-4细胞计数，设计9组，分别为空白组、对照组（PPⅦ浓度0 µmol/L）及加药组（PPⅦ浓度分别为0.25、0.5、1、1.5、2、2.5、5 µmol/L），每组设6个复孔，调整细胞密度为6×10^4^个/ml，每孔100 µl接种于96孔板，在板子边缘孔加入PBS防止边缘效应。置于细胞培养箱中培养24 h、48 h、72 h。每孔加入MTT溶液（50 mg/ml）20 µl，细胞培养箱中避光孵育4 h后加入MTT溶解液100 µl，培养箱中过夜，次日取出用酶标仪测570 nm、650 nm的吸光度值（A），*A*=A570−A650，细胞存活率＝（*A*药物组−*A*空白组）/（*A*对照组−*A*空白组）×100％。细胞增殖抑制率＝（1−细胞存活率）。

4. 流式细胞术检测细胞凋亡：取U2932、SUDHL-4细胞计数，设计4组，分别为对照组（PPⅦ浓度0 µmol/L）、加药组（PPⅦ浓度分别为0.25、0.5、1 µmol/L），调整细胞密度为2×10^5^个/ml，每孔1 ml接种于24孔板。用药处理48 h后收集细胞悬液，预冷PBS洗涤细胞2次，用400 µl 1X Annexin Ⅴ结合液重新悬浮细胞，设置单染PI管、单染FITC管、空白管及实验组管，先在单染FITC管及实验组管中加入5 µl Annexin Ⅴ-FITC染色液，轻轻混匀后于4 °C避光条件下孵育15 min。后在单染PI管及实验组管中加入8 µl PI染色液，轻轻混匀后于4 °C避光条件下孵育5 min，在流式细胞仪上检测细胞凋亡情况。

5. 流式细胞术检测细胞周期：取U2932、SUDHL-4细胞，用1640培养基饥饿细胞24 h，将细胞同步化。同上设计4组，调整细胞密度为2×10^5^个/ml，每孔1 ml接种于24孔板。用药处理24 h后收集细胞悬液，预冷PBS洗涤细胞2次，缓慢滴加预冷的乙醇，置于−20 °C冰箱1 h固定细胞。取出后预冷PBS洗涤细胞2次，加入Rnase A溶液20 µl，置于37 °C水浴锅中水浴30 min，离心弃去染液及PBS，加入400 µl PI染液重悬细胞，轻轻混匀后置于4 °C冰箱避光孵育30 min，在流式细胞仪上检测细胞周期。

6. Western blot法检测凋亡相关蛋白及周期相关蛋白的表达：取U2932、SUDHL-4细胞计数，同上设计4组，调整细胞密度为2×10^5^个/ml，每孔1 ml，接种于24孔板。用药处理48 h后收集细胞悬液，预冷PBS洗涤细胞2次，加入裂解试剂后置于冰上裂解，离心收集上清液。在上清液中加入相应体积的蛋白上样缓冲液，置于恒温金属浴，100 °C煮10 min使蛋白变性。在低温条件下以恒流250 mA将电泳后分离的蛋白转移至PVDF膜上，使用5％脱脂牛奶封闭2 h后分别加入相应一抗置于摇床1 h，后于4 °C冰箱过夜，次日孵育相应二抗置于摇床1 h，应用ECL试剂盒在化学发光成像仪中显影，内参选用GADPH，应用Image J软件进行条带分析。

7. 统计学处理：数据分析使用GraphPad Prism 8.0软件，结果以至少3个独立实验的*x±s*表示，多组间比较采用单因素方差分析，以*P*<0.05为差异具有统计学意义。

## 结果

1. PPⅦ对DLBCL细胞增殖活性的影响：加入不同浓度的PPⅦ处理24 h、48 h、72 h后，U2932、SUDHL-4细胞的增殖情况如图1所示。0.25、0.5、1、1.5、2、2.5、5 µmol/L PPⅦ处理U2932细胞24 h、48 h、72 h后，与对照组相比差异均有统计学意义（*P*值均<0.05）；0.5、1、1.5、2、2.5、5 µmol/L PPⅦ处理SUDHL-4细胞24 h、48 h、72 h后，与对照组相比差异均有统计学意义（*P*值均<0.05），0.25 µmol/L PPⅦ处理SUDHL-4细胞48 h、72 h后与对照组相比差异具有统计学意义（*P*<0.05）。

**图1:**
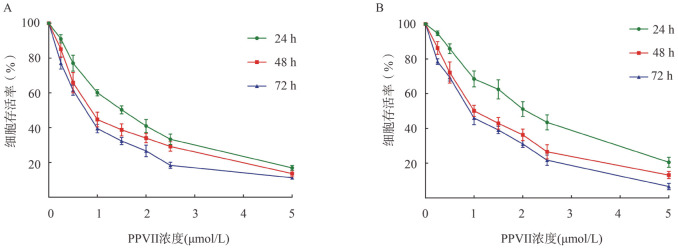
MTT法检测重楼皂苷Ⅶ（PPⅦ）对U2932（A）、SUDHL-4（B）细胞增殖活性的影响

2. PPⅦ对DLBCL细胞株凋亡的影响：加入不同浓度的PPⅦ处理48 h后，U2932、SUDHL-4细胞的凋亡情况如图2所示。结果显示：与对照组相比较，随着PPⅦ浓度增加，凋亡细胞占比明显增加。0.5、1µmol/L PPⅦ处理U2932细胞48 h后，凋亡率分别为（70.57±6.28）％、（35.80±1.44）％；0.5、1 µmol/L PPⅦ处理SUDHL-4细胞48 h后，凋亡率分别为（74.73±8.34）％、（59.97±9.96）％。提示PPⅦ可诱导U2932、SUDHL-4细胞凋亡，且具有浓度依赖性。

**图2:**
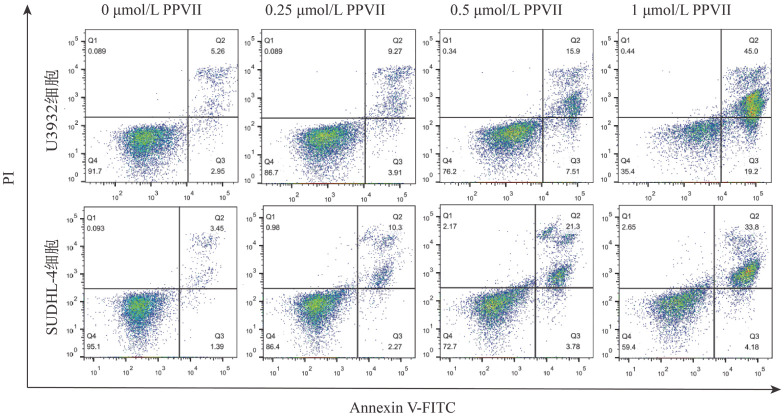
流式细胞术检测重楼皂苷Ⅶ（PPⅦ）对U2932、SUDHL-4细胞凋亡能力的影响

3. PPⅦ对DLBCL细胞株凋亡相关蛋白表达的影响：Western blot结果如图3所示，与对照组比较，0.5、1 µmol/L PPⅦ组抗凋亡蛋白Bcl-2的表达量明显下调，促凋亡蛋白Bax、Smac、Cleaved Caspase 3、Cleaved Caspase 7、Cleaved Caspase 9、P53的表达量明显上调（*P*值均<0.05），提示PPⅦ可通过调节凋亡相关蛋白的表达水平促进DLBCL细胞凋亡。

**图3:**
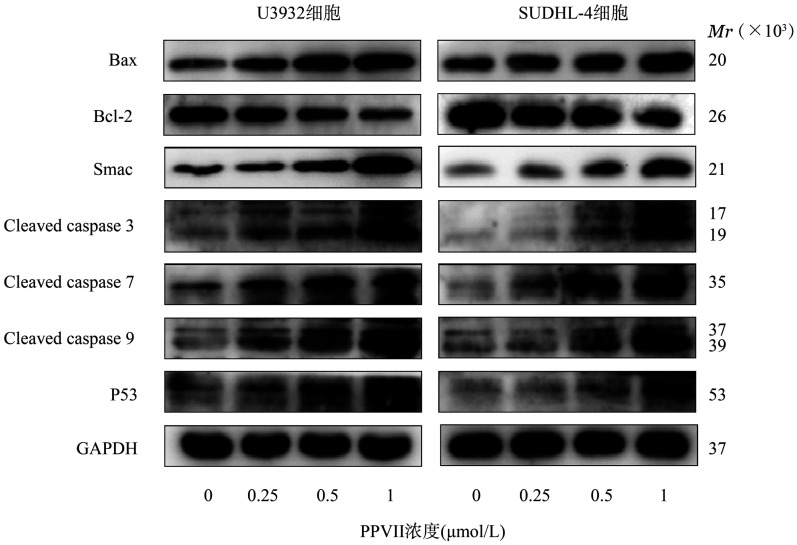
Western blot法检测U2932、SUDHL-4细胞经重楼皂苷Ⅶ（PPⅦ）不同浓度处理后凋亡相关蛋白表达水平的影响

4. PPⅦ对DLBCL细胞株细胞周期的影响：加入不同浓度的PPⅦ溶液24 h后，U2932、SUDHL-4细胞的细胞周期变化情况如图4所示。与对照组相比较，随着PPⅦ浓度增加，G_0_/G_1_期细胞增加，G_2_/M期细胞减少。提示PPⅦ可使U2932、SUDHL-4淋巴瘤细胞阻滞于G_0_/G_1_期，具有浓度依赖性。统计学结果提示：0.5、1 µmol/L PPⅦ处理U2932、SUDHL-4细胞24 h后，G_0_/G_1_期、G_2_/M期与对照组相比差异均具有统计学意义（*P*值均<0.05）。

**图4:**
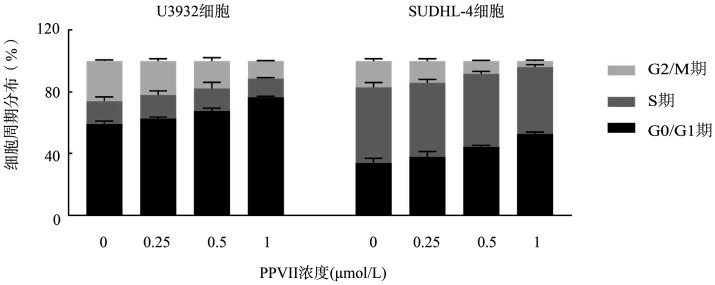
流式细胞术检测重楼皂苷Ⅶ（PPⅦ）对U2932、SUDHL-4细胞周期的影响

5. PPⅦ对DLBCL细胞株细胞周期相关蛋白表达的影响：Western blot结果如图5所示，与对照组比较，0.5、1 µmol/L PPⅦ组Survivin蛋白、细胞周期蛋白Cyclin D1及细胞周期蛋白依赖性激酶CDK4、CDK6的表达量明显下调（*P*值均<0.05），提示PPⅦ可通过调节周期相关蛋白的表达水平使细胞周期发生阻滞。

**图5:**
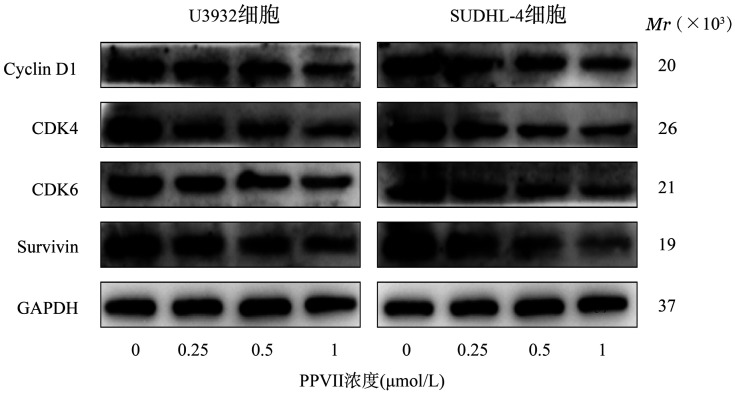
Western blot法检测重楼皂苷Ⅶ（PPⅦ）对U2932、SUDHL-4细胞周期相关蛋白表达水平的影响

## 讨论

DLBCL是最常见的非霍奇金淋巴瘤，属于高侵袭性B细胞淋巴瘤。目前的治疗方案R-CHOP等虽然可使其缓解率较前增加，但仍有部分患者存在疗效不佳、不良反应大，耐药等情况，开发新型药物提高缓解率具有重大意义[8]–[9]。PPⅦ是中药重楼中的主要活性成分之一，近年来许多研究发现，与传统化疗药物相比，具有低毒、安全、来源广泛等优势，已有研究发现其具有较好的抗肿瘤作用。本研究结果显示，PPⅦ能够有效抑制U2932、SUDHL-4细胞增殖、促进细胞凋亡，且与时间和浓度呈正相关，为PPⅦ的临床应用提供了初步的实验依据。

细胞凋亡是程序性细胞死亡的一种形式，可有效清除体内不需要或异常的细胞，其机制的失调是DLBCL发生发展的重要标志[10]。P53是一种肿瘤抑制因子，可与Bcl-2家族蛋白相互作用，导致促凋亡蛋白Bax的释放，引起线粒体膜通透性发生改变，进而启动一系列Caspase级联反应[11]。Caspase成员与多种肿瘤细胞凋亡密切相关，可分为启动子如Caspase 9和效应子如Caspase 3、Caspase 7等，Caspase酶原的活化是细胞凋亡的关键环节[12]；而Smac可以解除凋亡抑制蛋白[13]对Caspase家族的抑制，参与细胞凋亡的下游反应。本研究发现，PPⅦ可抑制抗凋亡蛋白Bcl-2的表达，增加促凋亡蛋白P53、Bax、Smac的表达，同时增加Cleaved Caspase 3、Cleaved Caspase 7、Cleaved Caspase 9蛋白表达，由此推测PPⅦ可能通过激活P53和Smac蛋白表达，启动Caspase级联反应，从而促进DLBCL细胞凋亡。

细胞周期受到细胞周期蛋白（Cyclin）、细胞周期蛋白依赖性激酶（CDK）等分子的严格调控[14]，其进展异常是影响DLBCL细胞增殖与分化的主要因素之一。研究显示，Survivin和Cyclin D1表达均具有周期性特征，可与CDK结合，调节G_1_/S期的转换[15]–[16]。本研究结果显示，PPⅦ可阻滞U2932、SUDHL-4细胞在G_0_/G_1_期，同时抑制Cyclin D1、CDK4、CDK6及Survivin蛋白的表达，表明PPⅦ可通过调节周期相关蛋白表达阻滞DLBCL的周期进展。

综上所述，PPⅦ可显著抑制DLBCL细胞增殖，促进细胞凋亡，并使细胞周期阻滞在G_0_/G_1_期。本研究将进一步完善PPⅦ抗DLBCL的体内实验，为PPⅦ抗肿瘤临床应用提供更多的理论和实验依据。
